# Optimized Solid Phase-Assisted Synthesis of Dendrons Applicable as Scaffolds for Radiolabeled Bioactive Multivalent Compounds Intended for Molecular Imaging

**DOI:** 10.3390/molecules19066952

**Published:** 2014-05-27

**Authors:** Gabriel Fischer, Björn Wängler, Carmen Wängler

**Affiliations:** 1 Biomedical Chemistry, Department of Clinical Radiology and Nuclear Medicine, Medical Faculty Mannheim of Heidelberg University, Mannheim 68167, Germany; E-Mail: Gabriel.Fischer@medma.uni-heidelberg.de; 2 Molecular Imaging and Radiochemistry, Department of Clinical Radiology and Nuclear Medicine, Medical Faculty Mannheim of Heidelberg University, Mannheim 68167, Germany; E-Mail: Bjoern.Waengler@medma.uni-heidelberg.de

**Keywords:** modular dendron synthesis, multimerization, radiolabeling, solid support

## Abstract

Dendritic structures, being highly homogeneous and symmetric, represent ideal scaffolds for the multimerization of bioactive molecules and thus enable the synthesis of compounds of high valency which are e.g., applicable in radiolabeled form as multivalent radiotracers for *in vivo* imaging. As the commonly applied solution phase synthesis of dendritic scaffolds is cumbersome and time-consuming, a synthesis strategy was developed that allows for the efficient assembly of acid amide bond-based highly modular dendrons on solid support via standard Fmoc solid phase peptide synthesis protocols. The obtained dendritic structures comprised up to 16 maleimide functionalities and were derivatized on solid support with the chelating agent DOTA. The functionalized dendrons furthermore could be efficiently reacted with structurally variable model thiol-bearing bioactive molecules via click chemistry and finally radiolabeled with ^68^Ga. Thus, this solid phase-assisted dendron synthesis approach enables the fast and straightforward assembly of bioactive multivalent constructs for example applicable as radiotracers for *in vivo* imaging with Positron Emission Tomography (PET).

## 1. Introduction

Dendritic structures, exhibiting highly branched and ideally well-defined and homogeneous structures, have extensively been studied as carriers for a large variety of bioactive compounds over several decades [1,2,3]. 

The standard procedure for the preparation of dendrimers is still the solution-phase synthesis as by this technique, very large dendritic structures can be synthesized on a large scale. However, this synthesis approach is often time-consuming due to the long reaction times required for a complete conversion of all functional groups and the cumbersome purification procedures that have to be implemented after each reaction step. 

An alternative is the synthesis of dendrimers using a solid support, which exhibits the advantages of comparatively fast reaction kinetics (for each synthesis step, reaction times of several hours per reaction step are required, whereas in case of solution phase dendrimers, reaction times of days to weeks have to be applied to obtain substances of high homogeneity) and very efficient purifications which are executed by simple washing of the resin. 

So far, several examples for the solid phase-assisted synthesis of dendrimers are available [4], including the syntheses of polyamidoamine (PAMAM) dendrimers [5,6,7], polylysine dendrimers [8,9,10], poly-amino acid dendrimers [11,12,13,14,15], aromatic polyethers [16,17,18,19] and polyurea-based dendrimers [20,21,22]. However, the vast majority of these syntheses apply definite building blocks, defining the structure of the resulting dendrimers and thus do not enable a modular dendritic molecular design. 

Such a modular dendrimer design can be of special interest if the dendrimers are to be used as drug carriers with a certain, optimal distance required between two drug moieties within the molecule. So was for example shown very recently that the distance between peptide moieties within a dendritic peptide multimer has a crucial influence on the achievable binding avidities of the multivalent peptides to their respective receptor [23]. 

Only very few examples of solid phase-supported syntheses of structurally flexible polylysine dendrimers have been described recently and in these systems, the structural flexibility was achieved by the introduction of different linkers [8,24]. To broaden this approach from the synthesis of dendritic drug carriers to dendron scaffolds applicable in the multimerization of bioactive molecules yielding multivalent compounds intended for an *in vivo* molecular imaging application, these dendrons also have to provide the opportunity to introduce a label such as a radionuclide. The optimal position for the introduction of this label is the focal point of the dendron to ensure a highly homogeneous and defined structure of the whole construct.

Such dendritic scaffolds, offering optimal properties such as a symmetrically branched and highly homogeneous structure while at the same time enabling a tailored molecular design with variable distances between surface functionalities, are an ideally suited platform for example for the synthesis of peptidic multimers. Such multivalent bioactive compounds have shown to be highly favorable radiotracers for the diagnostic imaging of different cancer types when radiolabeled and applied in *in vivo* imaging settings [23,25,26,27,28,29,30,31,32].

To improve the existing synthesis pathways towards dendritic structures which are highly flexible in molecular design, are accessible by solid phase-supported synthesis protocols and can be furthermore applied in multimerization reactions and molecular imaging settings, we intended to develop a synthesis route which should fulfill the following requirements: (*i*) a structure based on acid-amides as these are accessible by standard Fmoc solid phase peptide synthesis protocols enabling efficient syntheses and simple purifications while exhibiting a high *in vivo* stability and low immunogenicity; (*ii*) a high flexibility in molecular design which is important as these dendritic scaffolds should be applicable to the multimerization of peptides and is to be achieved by the use of structurally modular building blocks; (*iii*) the possibility to use oligoethyleneglycol (OEG) spacers as these structures are able to modulate the distance between the dendron surface functionalities, contribute to a higher hydrophilicity of the scaffold structures and are furthermore able to positively influence blood circulation half-lives and tumor accumulations [33]; (*iv*) the possibility to introduce a chelator for radiometal labeling or a functionality for biomolecule conjugation adjacent to the focal point of the dendron; and (*v*) an efficient synthesis giving the desired products (monovalent/multivalent maleimides up to maleimide hexadecimers applicable in biomolecule multimerization reactions) in ideally high efficiency and homogeneity. The surface functionalization of the dendrons with maleimides was chosen as the Michael addition reaction of maleimides and thiols was shown to be—compared to other available chemoselective click chemistry reactions—superior regarding the conjugation and thus multimerization of bioactive molecules because of quantitative reactions [29]. By the approach described here, dendrons being highly modular in structure and comprising up to 16 maleimide functional groups could be obtained and were shown to be applicable in subsequent multimerization click chemistry reactions with different thiol-bearing synthons. Furthermore, a chelator could easily be introduced adjacent to the focal point of the dendrons and the final multivalent products could be radiolabeled with ^68^Ga.

## 2. Results and Discussion

### 2.1. Dendron Scaffold Synthesis and Design

**Scheme 1:**
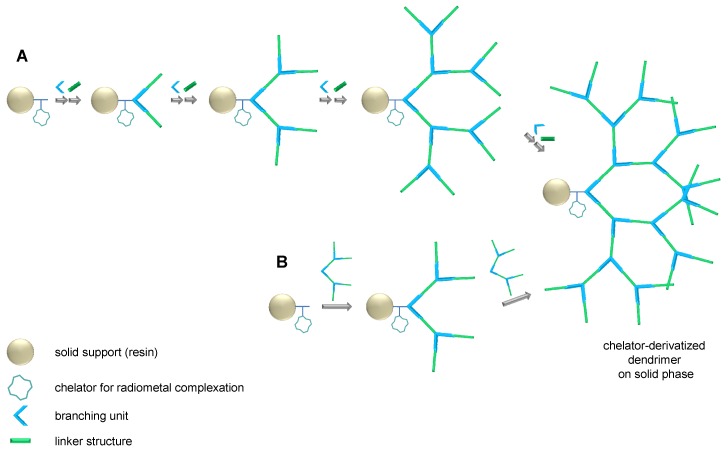
Schematic depiction of a divergent (**A**) *vs.* convergent (**B**) synthesis approach of dendritic scaffolds on the example of a dendron assembled on solid support. The radiometal labeling of the dendrons is enabled via the introduced chelator.

For the synthesis of dendritic structures, two different processes can be applied: a divergent or a convergent synthesis approach (Scheme 1). Convergent syntheses, building the dendron by conjugation of dendritic, comparatively large synthons in every synthesis step, enable an efficient assembly of the dendron in only few steps. In contrast, a divergent synthesis route assembles the dendron by stepwise conjugation of small building blocks and thus comprises a higher number of reaction steps but on the other hand enables a higher modularity of the dendron.

Hence, a divergent route was followed for this solid phase-assisted dendron synthesis approach as this allows for the use of different building blocks in every synthesis step, thus a maximal modular structure of the resulting dendritic scaffolds as well as tailored distances between two dendron surface functionalities. 

The dendron assembly on solid support thus comprises the following steps: (*i*) the coupling of an initial amino acid (used in order to obtain a functional group which can subsequently be used for a modification of the dendron with a chelator or another label or for the conjugation of the multimer); (*ii*) branching amino acids and/or linkers are conjugated until the complete dendritic structure is assembled on the resin; (*iii*) the derivatization of the amino groups on the surface of the dendron, enabling an efficient subsequent biomolecule conjugation; (*iv*) the cleavage of the whole construct from the solid support under acidic conditions; (*v*) analysis and/or purification of the obtained raw materials by HPLC. The multivalent and homogeneously functionalized dendrons can in the following be reacted with bioactive substances, yielding the respective multivalent biomolecules which can be used for a subsequent conjugation or radiolabeling and thus for *in vivo* imaging applications.

**Figure 1:**
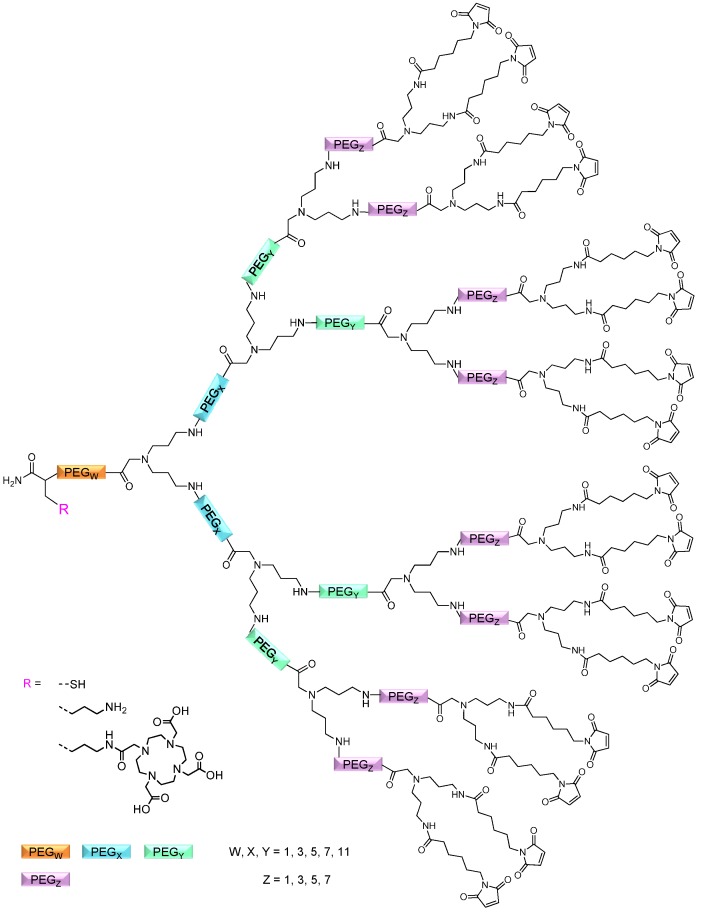
Schematic depiction of the general structure of the dendritic scaffolds synthesized on solid support. The dendrons comprise terminal maleimide functionalities for the efficient Michael addition of arbitrary thiol-bearing molecules, symmetrical branching units, oligoethyleneglycols of differing lengths for modular molecular design and tailored distances between two adjacent dendron surface functionalities as well as the possibility to introduce a chelator or thiol functionality for radiometal labeling or biomolecule conjugation, respectively.

Besides enabling a highly flexible synthesis and thus molecular structure, the principle design of the dendrons should follow the composition of conventionally synthesized PAMAM dendrons successfully used before for the synthesis of multivalent peptides [23,29]. These substances comprised—besides a highly symmetrical and homogeneous structure and up to 16 terminal maleimide functionalities on the outer shell—an OEG linker between the focal point of the dendron and the introduced chelator, a feature being advantageous for chelator conjugation, dendron assembly as well as radiolabeling [34] due to a less pronounced steric hindrance exerted by the branched structure. Furthermore, OEGs inserted between branching synthons were expected to also have a positive influence on dendron assembly and synthesis efficiency and were thus included in the synthesis rationale. In Figure 1, the schematic structure of the synthesized dendron scaffolds is depicted. 

#### 2.1.1. Influence of OEG Linkers on Product Purities and Yields

**Scheme 2:**
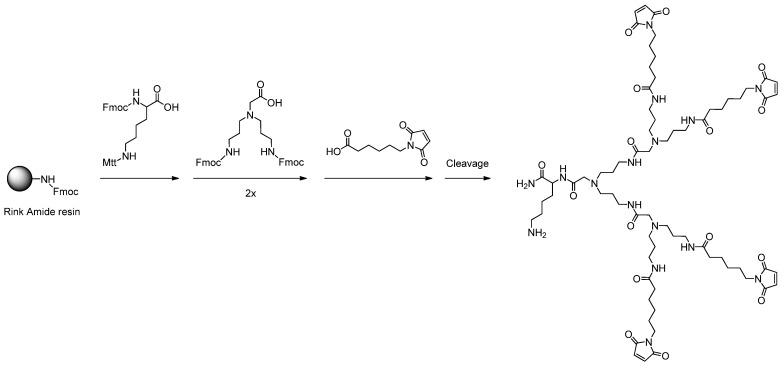
Schematic depiction of the reaction pathway for the synthesis of non-OEG-comprising dendritic structures on the example of a maleimide tetramer.

To obtain dendritic structures applicable as scaffolds for the multimerization of arbitrary thiol-bearing bioactive molecules while also enabling the introduction of a chelator for radiometal labeling, the first synthesis attempts comprised the conjugation of a lysine to the solid support and the subsequent conjugation of the branching units. For the first synthesis experiments, a standard rink amide resin with a loading of 0.74–0.79 mmol/g was used as solid support and the conjugation reactions were performed using standard Fmoc solid phase peptide synthesis protocols and reactant excesses of four equivalents of amino acid per amino functionality to be derivatized. As branching unit, *N*,*N*-bis(*N'*-Fmoc-3-aminopropyl)-glycine potassium hemisulfate (APG) was used as this amino acid in contrast to a branching lysine results in symmetric dendritic structures without multiple stereo centers (Scheme 2). Finally, maleimido hexanoic acid was conjugated to the terminal amino functionalities, yielding the respective multivalent maleimides.

The resulting dimers and tetramers synthesized by this procedure could however be obtained in only low purities of the cleaved raw materials. These findings can be attributed to the steric hindrance exerted by the relatively bulky structures and their small distances to one another, impeding efficient conjugation reactions. 

**Figure 2:**
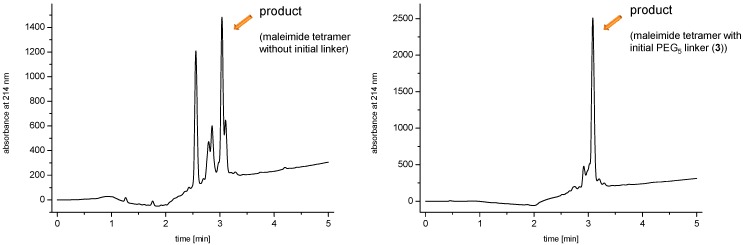
Analytical HPLC traces of the raw materials of a tetravalent maleimide synthesized on a standard rink amide resin comprising no OEG linker (**left**) or on a low-loading resin implementing a PEG_5_-linker (**right**) between the initially conjugated lysine and the focal point of the dendron.

In the following was thus used the same conjugation chemistry but a low-loading rink amide resin with a loading of only 0.21–0.23 mmol/g. Furthermore, an OEG linker was implemented between the initially conjugated lysine or cysteine amino acid and the focal point of the dendrons. These measures resulted in significantly improved raw product purities of 83%*vs.* 31% for tetramers (Figure 2).

High purities of the cleaved raw materials are a prerequisite for a straightforward synthesis and also for reasonable isolated product yields and were thus in the following used as a measure for the synthesis efficiency. 

**Figure 3:**
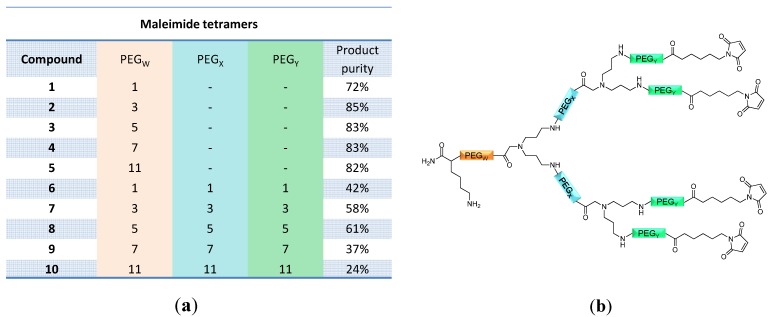
Observed product purities of the cleaved raw materials (**a**); and schematic depiction of the structures of the tetravalent maleimides **1**–**10** (**b**). Product purities were determined by analytical HPLC.

**Figure 4:**
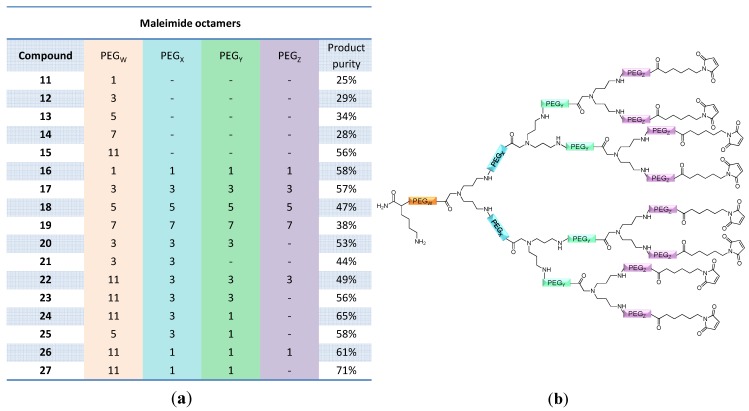
Obtained product purities of the cleaved raw materials (**a**); and schematic depiction of the structures of the octavalent maleimides **11**–**27** (**b**). Product purities were determined by analytical HPLC.

In the following, the length of the OEG linker inserted between the initial amino acid and the focal point of the dendrons (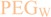 linker, Figure 3 and Figure 4) was systematically optimized for the tetrameric and octameric maleimides using OEGs of different lengths (PEG_1_, PEG_3_, PEG_5_, PEG_7_ and PEG_11_; OEG linkers are denoted as “PEGs” due to their trade names). For the tetramers, the length of the applied OEG did not seem to have a crucial influence on the achievable product purities after cleavage as long as it exceeded the length of a PEG_1_ (**1**–**5**, Figure 3). 

In contrast to these findings for tetramers, the highest maleimide octamer raw product purities obtained after cleavage were found using a PEG_11_ as initial 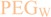 linker (**11**–**15**, Figure 4). Thus, the importance to initially implement a long linker structure before assembling the dendron is of higher importance for larger constructs which can be attributed to their larger size and thus steric demand necessitating a larger distance to the resin for an efficient synthesis. 

To evaluate if the reaction efficiency can be further optimized introducing additional OEG linkers in other positions of the dendrons (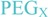, 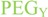 and 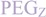 linkers, Figure 4), OEGs of different lengths were in the following not only implemented between the initial amino acid and the focal point of the dendrons (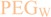 linker), but also after each branching amino acid. Systematically investigating the influence of these additional OEG linkers, it was found that the tetrameric maleimide dendrons could not profit from the introduction of further (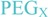 and 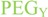) linkers (**6**–**10**, Figure 3). 

**Figure 5:**
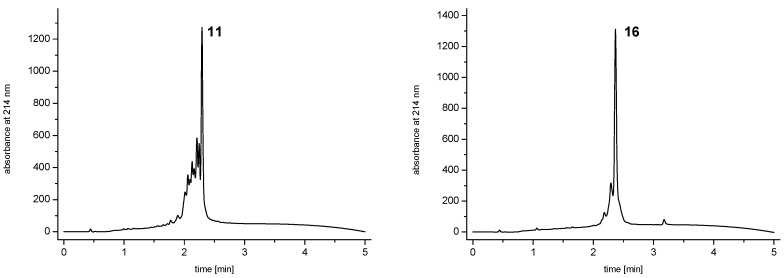
Analytical HPLC traces of the raw materials of octavalent maleimides synthesized comprising only one initial PEG_1_ (**11**, left) or PEG_1_-linkers in each possible position of the dendron (**16**, right).

In contrast to these findings for tetramers—and as shown before for the initially conjugated 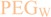 linker—the introduction of additional OEG (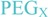, 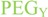 and 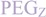) linkers was however able to result in a considerable increase of product formation in case of octavalent maleimides (**16**–**27**, Figure 4). The positive influence however strongly depended on the linker lengths used. So could for example be shown that the product purities of the raw materials increased significantly when inserting an additional linker after each branching amino acid (comparing the raw product purities of **11** and **16** (Figure 5), **12** and **17**, **13** and **18** as well as **14** and **19**) but that PEG_1_-linkers gave the best results when using the same linker length in every position (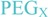, 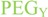 and 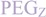; comparing product purities of **16**, **17**, **18** and **19**). 

This negative effect of using long linkers in each position of the dendron scaffold may be attributable to the strongly increased flexibility when using long OEGs which on the one hand decreases the steric hindrance for the following reactions but on the other hand obviously also increases entropic or coiling effects hampering an efficient conjugation of following building blocks thus outbalancing the positive effects of linker introduction.

Overall, the highest reaction efficiencies were observed for octavalent maleimides containing a PEG_11_ linker between the initial amino acid and the focal point of the dendron and additional PEG_1_ linkers after the branching units. Interestingly, the introduction of an OEG linker before conjugating the surface maleimide functionalities did not give improved, but even deteriorated results (comparing results obtained for **22** and **23** as well as **26** and **27**). 

Taken together, these results indicate that not only the steric hindrance between dendritic scaffold and resin can hamper the efficient conjugation reactions of building blocks but also the steric hindrance which is exerted by the dendritic structure itself if exceeding a certain size.

**Figure 6:**
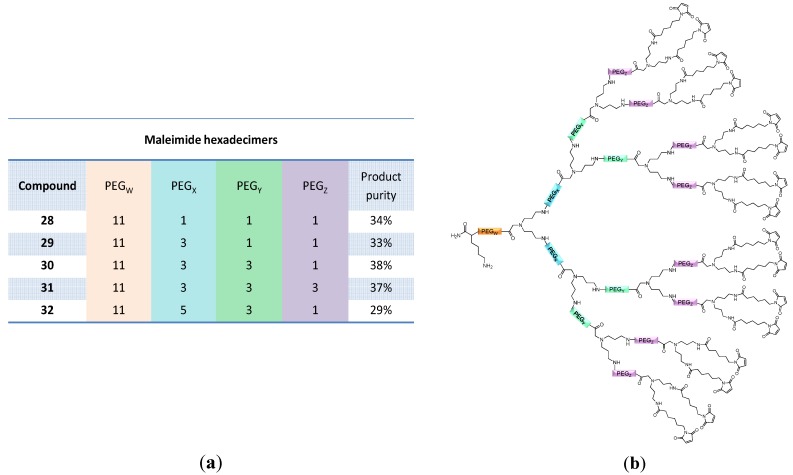
Obtained product purities of the cleaved raw materials (a); and schematic depiction of the structures of hexadecimeric maleimides **28**–**32** (**b**). Product purities were determined by analytical HPLC.

Taking these findings into account, maleimide hexadecimers were synthesized in the following and could be obtained in purities of the raw materials of up to 38% (Figure 6). Although these raw product purities at first glance seem to be rather low, they are in a comparable range to those observed during solution-phase dendron syntheses taking the following consideration into account: a corresponding solution-phase multi-step dendron synthesis approach produces similar amounts of by-products during each single synthesis step that are however removed after each reaction. In case of a solid phase-assisted synthesis, the formed by-products are not removed after each synthesis step, but only non-reacted materials. This results in lower overall purification efforts but a higher purification complexity of the final products during the solid phase dendron synthesis, however not significantly influencing the obtainable overall product purities. 

#### 2.1.2. Optimization of Other Reaction Parameters

Besides the molecular design which was shown to exert a significant influence on reaction efficiencies and thus product yields, also other parameters turned out to be important to obtain optimal synthesis results. It could be observed that—with an increasing number of branching units within the dendrons and thus an increasing steric demand of the constructs—the conjugation times for the following building blocks had to be prolonged and furthermore depended on the structure of the synthon to be coupled. So could be shown that for linear building blocks such as OEG linkers and maleimido hexanoic acid, the optimal results could be obtained extending the reaction times from 30 min over 45 min and 60 min to 120 min, depending on the number of branching units and the position within the dendritic structure. In case of the branching amino acid *N*,*N*-bis(*N*'-Fmoc-3-aminopropyl)-glycine potassium hemisulfate (APG), generally longer reaction times had to be applied, increasing from 60 min for the first branching APG unit over 120 min for the second APG unit to reaction times over night for all following APG units to obtain optimal results. Longer reaction times than the mentioned ones did however not result in improved product purities. 

Besides reaction times, the reactant excess used also has a considerable influence on the achievable reaction efficiencies. Astonishingly, the best results were not obtained for a reactant excess of four equivalents of building block per reactive functionality as it is generally applied in Fmoc solid phase peptide synthesis. In contrast, two equivalents turned out to be optimal. Thus, for example it could be shown that during the synthesis of **17** the reactant excess of four equivalents of building block per reactive functionality resulted in a raw product purity of 41%, whereas 3, 2 and 1.5 equivalents gave the product in improved purities of 51%, 57% and 52%, respectively. A possible explanation for these results might be the long reaction times applied during the dendron syntheses that could—in combination with the higher reactant excesses—result in a higher probability of side reactions and thus decreased product purities compared to standard peptide syntheses.

All in all, it could be demonstrated that the synthesis of even comparably large multivalent structures of up to hexadecimers could be accomplished on solid support. Varying the structural design of the dendrons, the products could be obtained in reasonable yields and although showing preparation optima for certain structures, also deviant structures could be successfully synthesized. Furthermore, the preparation times for the solid phase-dendrons were between 1 and 3 days—depending on the generation of the dendritic structure—which is considerably faster than the equivalent dendron synthesis in solution. So could e.g., octameric structures be completely assembled on solid support within less than 24 h using this procedure whereas the corresponding PAMAM dendrons have to be synthesized over a reaction time of 30 days (over all reaction steps), not including the time required for the mandatory purifications, to obtain highly homogeneous products [35]. 

### 2.2. Applicability of the Multivalent Maleimides in Multimerization Reactions

**Figure 7:**
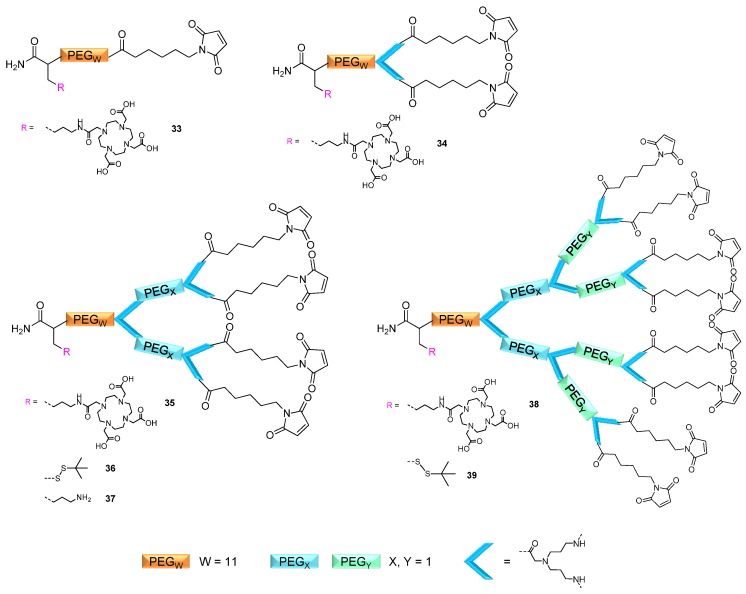
Schematic depiction of the synthesized mono/multivalent maleimides **33**–**39** comprising either a *t*Bu-thio-protected thiol functionality for bioconjugation or a DOTA chelator for radiometal labeling.

To show the applicability of the synthesized dendritic multivalent maleimide scaffold structures in the multimerization of thiol-bearing synthons and subsequent radiolabeling, different scaffolds were synthesized according to the afore-mentioned optimized reaction conditions. In principle, different multivalent structures could be of interest for an application in *in vivo* imaging. For example, multivalent peptides were shown to be highly affine imaging agents in tumor diagnosis [29,36] and DOTA (1,4,7,10-tetraazacyclododecane-1,4,7,10-tetraacetic acid) multimers could be interesting synthons for a conjugation to a targeting biomolecule to achieve improved specific activities during radiolabeling [35]. Thus, different mono-, di-, tetra- and octameric maleimide scaffolds (**33**–**39**, Figure 7) were synthesized: mono/multivalent maleimides comprising either a *t*Bu-thio-protected thiol functionality (which can be reductively deprotected and subsequently reacted with maleimide-derivatized biomolecules) or a DOTA moiety for radiometal labeling and subsequent *in vivo* PET imaging.

Concerning the introduction of DOTA into the dendritic scaffolds, it was observed that only slightly higher product purities of the raw materials (~4%) could be obtained when conjugating tris-*t-*Bu-DOTA directly after the lysine and before the dendron was assembled. Interestingly, also the inverse order, first synthesizing the dendritic scaffold before conjugating the chelator, gave the desired products in almost identical yields although a decrease in DOTA-conjugation efficiency was assumed to be exerted by the steric hindrance of the dendritic structure. 

The maleimide-comprising scaffolds to be applied in the following multimerization reactions were obtained in comparably low isolated yields between 2.9% and 17.0% after HPLC purification although the product purities of the raw materials after cleavage were adequate (between 53% and 78%). Furthermore, the observed isolated yields did not depend on dendron size or complexity (e.g., higher yields for di- and tetramers and lower yields for octamers would have indicated a loss of material during HPLC purification increasing with the size of the constructs due to growing interactions with the column material). Thus, a possible explanation for this effect of relatively low isolated yields could be an incomplete cleavage of the products from the solid support although even prolonged cleavage times did not give improved results. Another possibility might be that not all functional amides on the resin are sufficiently accessible to participate in chemical reactions as it was observed that even the mass of the isolated raw materials considerably deviated from the substance amounts that were estimated from the specified theoretical loading of the resin. To further substantiate this latter assumption, the resin was weighed before and after the assembly of the multivalent systems. By these experiments, it was e.g., found during the synthesis of **36** that only maximally ⅔ of the theoretical mass increase could be observed after the dendron assembly, implying an insufficient accessibility of the amides on the resin to chemical syntheses which limits the isolated product yields.

**Scheme 3:**
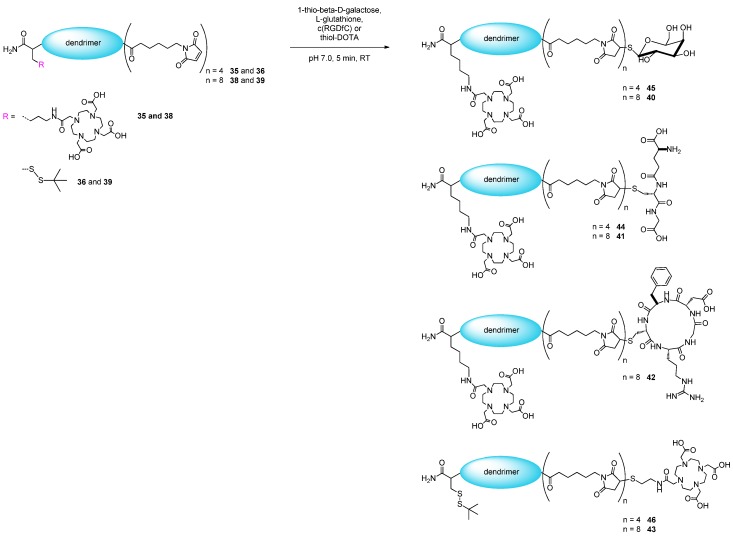
Schematic depiction of the reaction of the multivalent maleimides **35**, **36**, **38** and **39** with 1-thio-β-D-galactose, L-glutathione, c(RGDfC) and thiol-DOTA, giving the respective multivalent compounds **40**–**46**.

The tetravalent and octavalent maleimides **35**, **36**, **38** and **39** (Figure 7) were in the following reacted with different model thiols (1-thio-β-D-galactose, L-glutathione, c(RGDfC) and thiol-DOTA). The respective multivalent compounds (galactose_8_-DOTA (**40**), L-glutathione_8_-DOTA (**41**), c(RGDfC)_8_-DOTA (**42**), DOTA_8_-SS-*t*Bu (**43**), L-glutathione_4_-DOTA (**44**), galactose_4_-DOTA (**45**) and DOTA_4_-SS-*t*Bu (**46**)) (Scheme 3) were obtained in isolated yields of 18.1%–45.2% after HPLC purification, depending on the conjugated thiol-bearing synthon. 

Another observation was that the conjugation of the thiol-bearing synthons to the multivalent maleimides could be performed in solution after cleavage of the dendritic systems from the solid support and purification of the scaffolds, but also on solid support, thus further reducing the synthesis efforts for the multivalent constructs. This was exemplarily studied on the tetravalent compounds **44**, **45** and **46** which were synthesized partly in solution as described before (solid phase synthesis of the thiol- and DOTA-derivatized tetravalent maleimide scaffolds, cleavage, purification and conjugation of the respective thiols in solution) or completely on solid support (including the final conjugation reactions of the thiols to the maleimides before the products are cleaved from the resin). In both cases, an absolutely comparable product formation was observed (overall raw product purities of 69%–70% for **44**, 64%–67% for **45** and 56% for **46**), deviating only by up to 3% between both reaction pathways. This can be attributed to the quantitative conjugation of the thiols to the maleimides both in solution and on solid phase, meaning that the assembly of the dendron is the yield-limiting part of the synthesis. 

These results show the applicability of the multivalent maleimides synthesized on solid support for the multimerization of even structurally different building blocks.

### 2.3. ^68^Ga-Radiolabeling of the Octavalent Substances 40–43

As the aim of this study was to synthesize dendritic scaffolds that are not only applicable for the multimerization of varying thiol-bearing bioactive molecules but also for radiolabeling to be useful as radiotracers for *in vivo* imaging, the synthesized model octamers **40**–**43** were finally radiolabeled with ^68^Ga using the fractioned elution methodology of ^68^Ge/^68^Ga generators [37] and standard ^68^Ga-labeling conditions [23] to show the radiolabeling to be feasible. Of course, especially when using rather large dendritic structures exhibiting high molecular weights and thus comparatively slow *in vivo* pharmacokinetics for molecular imaging purposes, the use of other radiometal nuclides such as ^64^Cu could be more appropriate but depends on the actual *in vivo* pharmacokinetics of the used multimer.

The ^68^Ga-radiolabeling of the model octamers **40**–**43** proceeded efficiently although not all products could be isolated in sufficient purities. For **41**, a significant decomposition of the product could be observed directly after radiolabeling which could be decelerated but not completely suppressed by the addition of an excess of ascorbic acid. Thus, the observed fragmentation might be a result of radiolysis of the glutathione building block. In contrast to this, the ^68^Ga-labeling of **40**, **42** and **43** gave the radiolabeled products in high radiochemical yields and purities of ≥ 95% directly after labeling as well as high specific activities (up to 147 GBq/µmol for **40**, up to 55 GBq/µmol for **42** and up to 163 GBq/µmol for **43**) using starting activities of ~260 MBq ^68^Ga^3+^.

## 3. Experimental

### 3.1. General Information

All commercially available chemicals were of analytical grade and used without further purification. Resins for solid-phase synthesis, coupling reagents, Fmoc-protected amino acids, Fmoc-NH-PEG_1_-COOH and Fmoc-NH-PEG_3_-COOH were purchased from NovaBiochem (Schwalbach, Germany), 1-thio-β-D-galactose sodium salt, Fmoc-NH-PEG_5_-COOH, Fmoc-NH-PEG_7_-COOH and Fmoc-NH-PEG_11_-COOH were obtained from Iris Biotech (Marktredwitz, Germany), 6-maleimidohexanoic acid and L-glutathione (reduced) were purchased from Sigma Aldrich (Schnelldorf, Germany), *N*,*N*-bis(*N*'-Fmoc-3-aminopropyl)-glycine potassium hemisulfate (APG) was purchased from PolyPeptide (Strasbourg, France) and tris-*t-*Bu-DOTA was obtained from CheMatech (Dijon, France). For analytical and semipreparative HPLC chromatography, a Dionex UltiMate 3000 system (Idstein, Germany) equipped with a Gabi-Star (Raytest, Straubenhardt, Germany) radioactivity detector was used together with a Chromolith Performance (RP-18e, 100–4.6 mm, Merck, Darmstadt, Germany) and a Chromolith SemiPrep (RP-18e, 100–10 mm, Merck) column, operated with a flow of 4 mL/min. ESI (Electrospray Ionization) and MALDI (Matrix-Assisted Laser Desorption/Ionization) spectra were obtained using a Finnigan MAT95Q (Dreieich, Germany) and a Bruker Daltonics Microflex (Bremen, Germany) spectrometer together with gentisic or sinapic acid as matrix substances, respectively. The conjugation of tris-*t-*Bu-DOTA to the ε-amino function of lysine during solid phase synthesis was performed as described before [38]. c(RGDfC) and thiol-DOTA were synthesized as previously described [29,39].

### 3.2. General Procedure for the Solid Phase-Assisted Synthesis of Dendron Scaffolds

The dendron scaffolds were synthesized on solid support by standard Fmoc solid-phase peptide synthesis using an amino acid excess of 2 eq. per functionality and a standard conjugation time of 30 min, a commercially available standard Rink Amide resin (for compounds **6**–**10**) or low-loading NovaPEG Rink Amide resin (for compounds **1**–**5** and **11**–**39**), *N*_α_-Fmoc-amino acids, Fmoc-*N*_ω_-PEG-amino acids and *N*,*N*-bis(*N*'-Fmoc-3-aminopropyl)-glycine potassium hemisulfate (APG) as branching amino acid. The synthesized dendritic scaffolds were cleaved from the solid support by incubation with a mixture of TFA (trifluoroacetic acid)/TIS (triisopropylsilane)/H_2_O (95:2.5:2.5) for 60 min if the scaffolds did not contain a protected DOTA building block or 2 h if they contained tris-*t*Bu-DOTA, were suspended in diethyl ether and analyzed/purified by HPLC after drying. The products were isolated as white solids or colorless oils after lyophilization. Reaction conditions which deviate from the standard coupling conditions, gradients used for HPLC analysis/purification and analytical data for each compound are given below. 

*H_2_N-CO-Lys-PEG_1_-Mal_4_* **1**: Reaction times for conjugation of APG-1: 1 h, APG-2: 2 h, 6-maleimidohexanoic acid: 1 h; gradient: 10%–50% MeOH + 0.1% formic acid (FA) in 5 min, R_t_ = 3.4 min; ESI-MS (*m/z*) for [M+3H]^3+^ (calculated): 526.31 (526.31); [M+2H]^2+^ (calculated): 788.96 (788.96); [M+2Na]^2+^ (calculated): 810.94 (810.94). 

*H_2_N-CO-Lys-PEG_3_-Mal_4_* **2**: Reaction times for conjugation of APG-1: 1 h, APG-2: 2 h, 6-maleimidohexanoic acid: 1 h; gradient: 10%–50% MeOH + 0.1% FA in 5 min, R_t_ = 3.5 min; ESI-MS (*m/z*) for [M+3H]^3+^ (calculated): 560.33 (560.33); [M+2H]^2+^ (calculated): 839.99 (839.99); [M+H+Na]^2+^ (calculated): 850.98 (850.98); [M+2Na]^2+^ (calculated): 861.97 (861.97); [M+Na+K]^2+^ (calculated): 869.96 (870.03). 

*H_2_N-CO-Lys-PEG_5_-Mal_4_* **3**: Reaction times for conjugation of APG-1: 1 h, APG-2: 2 h, 6-maleimidohexanoic acid: 1 h; gradient: 10%–50% MeOH + 0.1% FA in 5 min, R_t_ = 3.7 min; MALDI-MS (*m/z*) for [M+H]^+^ (calculated): 1766.55 (1767.03); [M+Na]^+^ (calculated): 1788.98 (1789.01); [M+K]^+^ (calculated): 1805.58 (1805.12). 

*H_2_N-CO-Lys-PEG_7_-Mal_4_* **4**: Reaction times for conjugation of APG-1: 1 h, APG-2: 2 h, 6-maleimidohexanoic acid: 1 h; gradient: 10%–50% MeOH + 0.1% FA in 5 min, R_t_ = 3.8 min; ESI-MS (*m/z*) for [M+3H]^3+^ (calculated): 619.04 (619.03); [M+2H]^2+^ (calculated): 928.05 (928.04); [M+H+Na]^2+^ (calculated): 939.04 (939.04); [M+2Na]^2+^ (calculated): 950.03 (950.03); [M+Na+K]^2+^ (calculated): 958.02 (958.08). 

*H_2_N-CO-Lys-PEG_11_-Mal_4_* **5**: Reaction times for conjugation of APG-1: 1 h, APG-2: 2 h, 6-maleimidohexanoic acid: 1 h; gradient: 10%–50% MeOH + 0.1% FA in 5 min, R_t_ = 4.1 min; MALDI-MS (*m/z*) for [M+H]^+^ (calculated): 2031.36 (2031.19); [M+Na]^+^ (calculated): 2053.29 (2053.17). 

*H_2_N-CO-Lys-(PEG_1_)_3_-Mal_4_* **6**: Reaction times for conjugation of APG-1: 1 h, PEG_X_: 45 min, APG-2: 2 h, PEG_Y_: 1 h, 6-maleimido-hexanoic acid: 1h; gradient: 20%–55% MeOH + 0.1% FA in 5 min, R_t_ = 2.9 min; ESI-MS (*m/z*) for [M+3H]^3+^ (calculated): 816.46 (816.46); [M+2H+Na]^3+^ (calculated): 823.78 (823.78). 

*H_2_N-CO-Lys-(PEG_3_)_3_-Mal_4_* **7**: Reaction times for conjugation of APG-1: 1 h, PEG_X_: 45 min, APG-2: 2 h, PEG_Y_: 1 h, 6-maleimido-hexanoic acid: 1 h; gradient: 20%–55% MeOH + 0.1% FA in 5 min, R_t_ = 3.8 min; ESI-MS (*m/z*) for [M+4H]^4+^ (calculated): 791.21 (791.21); [M+Na+3H]^4+^ (calculated): 796.71 (796.71); [M+2Na+2H]^4+^ (calculated): 802.20 (802.20); [M+4Na]^4+^ (calculated): 813.20 (813.19); [M+3H]^3+^ (calculated): 1054.61 (1054.61); [M+Na+2H]^3+^ (calculated): 1061.94 (1061.94); [M+H+2Na]^3+^ (calculated): 1069.27 (1069.27); [M+3Na]^3+^ (calculated): 1076.60 (1076.60). 

*H_2_N-CO-Lys-(PEG_5_)_3_-Mal_4_* **8**: Reaction times for conjugation of APG-1: 1 h, PEG_X_: 45 min, APG-2: 2 h, PEG_Y_: 1h, 6-maleimido-hexanoic acid: 1 h; gradient: 20%–55% MeOH + 0.1% FA in 5 min, R_t_ = 4.3 min; MALDI-MS (*m/z*) for [M+H]^+^ (calculated): 3779.96 (3778.20). 

*H_2_N-CO-Lys-(PEG_7_)_3_-Mal_4_* **9**: Reaction times for conjugation of APG-1: 1 h, PEG_X_: 45 min, APG-2: 2 h, PEG_Y_: 1 h, 6-maleimido-hexanoic acid: 1 h; gradient: 20%–55% MeOH + 0.1% FA in 5 min, R_t_ = 4.8 min; ESI-MS (*m/z*) for [M+5H+Na]^6+^ (calculated): 736.93 (736.93); [M+4H+2Na]^6+^ (calculated): 740.59 (740.59); [M+3H+3Na]^6+^ (calculated): 744.43 (744.26); [M+5H]^5+^ (calculated): 879.72 (879.72); [M+4H+Na]^5+^ (calculated): 884.12 (884.11); [M+3H+2Na]^5+^ (calculated): 888.91 (888.51). 

*H_2_N-CO-Lys-(PEG_11_)_3_-Mal_4_* **10**: Reaction times for conjugation of APG-1: 1 h, PEG_X_: 45 min, APG-2: 2 h, PEG_Y_: 1 h, 6-maleimidohexanoic acid: 1 h; gradient: 0%–100% MeCN + 0.1% TFA in 5 min, R_t_ = 3.2 min; ESI-MS (*m/z*) for [M+7H]^7+^ (calculated): 804.76 (804.76); [M+6H+Na]^7+^ (calculated): 807.9 (807.9); [M+5H+2Na]^7+^ (calculated): 811.04 (811.04); [M+6H]^6+^ (calculated): 938.72 (938.72); [M+5H+Na]^6+^ (calculated): 942.39 (942.39); [M+4H+2Na]^6+^ (calculated): 946.05 (946.05). 

*H_2_N-CO-Lys-PEG_1_-Mal_8_* **11**: Reaction times for conjugation of APG-1: 1 h, APG-2: 2 h, APG-3: overnight, 6-maleimido-hexanoic acid: 2 h; gradient: 30%–55% MeOH + 0.1% FA in 5 min, R_t_ = 2.0 min; MALDI-MS (*m/z*) for [M+H]^+^ (calculated): 3033.76 (3033.75). 

*H_2_N-CO-Lys-PEG_3_-Mal_8_* **12**: Reaction times for conjugation of APG-1: 1 h, APG-2: 2 h, APG-3: overnight, 6-maleimido-hexanoic acid: 2h; gradient: 30%–55% MeOH + 0.1% FA in 5 min, R_t_ = 2.0 min; ESI-MS (*m/z*) for [M+5H]^5+^ (calculated): 627.97 (627.77); [M+4H]^4+^ (calculated): 784.46 (784.46); [M+3H]^3+^ (calculated): 1046.28 (1045.61). 

*H_2_N-CO-Lys-PEG_5_-Mal_8_* **13**: Reaction times for conjugation of APG-1: 1 h, APG-2: 2 h, APG-3: overnight, 6-maleimido-hexanoic acid: 2 h; gradient: 30%–55% MeOH + 0.1% FA in 5 min, R_t_ = 2.0 min; MALDI-MS (*m/z*) for [M+H]^+^ (calculated): 3224.24 (3223.87). 

*H_2_N-CO-Lys-PEG_7_-Mal_8_* **14**: Reaction times for conjugation of APG-1: 1 h, APG-2: 2 h, APG-3: overnight, 6-maleimido-hexanoic acid: 2 h; gradient: 30%–55% MeOH + 0.1% FA in 5 min, R_t_ = 1.9 min; MALDI-MS (*m/z*) for [M+H]^+^ (calculated): 3312.95 (3311.93). 

*H_2_N-CO-Lys-PEG_11_-Mal_8_* **15**: Reaction times for conjugation of APG-1: 1h, APG-2: 2h, APG-3: overnight, 6-maleimidohexanoic acid: 2h; gradient: 20%–50% MeOH + 0.1% FA in 5 min, R_t_ = 3.5 min; MALDI-MS (*m/z*) for [M+H]^+^ (calculated): 3488.57 (3488.03). 

*H_2_N-CO-Lys-(PEG_1_)_4_-Mal_8_* **16**: Reaction times for conjugation of APG-1: 1 h, PEG_X_: 45 min, APG-2: 2 h, PEG_Y_: 1 h, APG-3: overnight, PEG_Z_: 2 h, 6-maleimidohexanoic acid: 2 h; gradient: 30%–55% MeOH + 0.1% FA in 5 min, R_t_ = 2.4 min; MALDI-MS (*m/z*) for [M+H]^+^ (calculated): 5071.36 (5064.79); [M+2H]^2+^ (calculated): 2533.63 (2532.90). 

*H_2_N-CO-Lys-(PEG_3_)_4_-Mal_8_* **17**: Reaction times for conjugation of APG-1: 1 h, PEG_X_: 45 min, APG-2: 2 h, PEG_Y_: 1 h, APG-3: overnight, PEG_Z_: 2 h, 6-maleimidohexanoic acid: 2 h; gradient: 30%–55% MeOH + 0.1% FA in 5 min, R_t_ = 3.5 min; ESI-MS (*m/z*) for [M+7H]^7+^ (calculated): 943.12 (943.12); [M+Na+6H]^7+^ (calculated): 946.41 (946.26). 

*H_2_N-CO-Lys-(PEG_5_)_4_-Mal_8_* **18**: Reaction times for conjugation of APG-1: 1 h, PEG_X_: 45 min, APG-2: 2 h, PEG_Y_: 1 h, APG-3: overnight, PEG_Z_: 2 h, 6-maleimidohexanoic acid: 2 h; gradient: 30%–55% MeOH + 0.1% FA in 5 min, R_t_ = 4.1 min; MALDI-MS (*m/z*) for [M+H]^+^ (calculated): 7946.57 (7938.58); [M+2H]^2+^ (calculated): 3962.96 (3958.80). 

*H_2_N-CO-Lys-(PEG_7_)_4_-Mal_8_* **19**: Reaction times for conjugation of APG-1: 1 h, PEG_X_: 45 min, APG-2: 2 h, PEG_Y_: 1 h, APG-3: overnight, PEG_Z_: 2 h, 6-maleimidohexanoic acid: 2 h; gradient: 30%–55% MeOH + 0.1% FA in 5 min, R_t_ = 4.7 min; MALDI-MS (*m/z*) for [M+H]^+^ (calculated): 9283.40 (9275.47); [M+2H]^2+^ (calculated): 4626.83 (4619.19). 

*H_2_N-CO-Lys-(PEG_3_)_3_-Mal_8_* **20**: Reaction times for conjugation of APG-1: 1 h, PEG_X_: 45 min, APG-2: 2 h, PEG_Y_: 1 h, APG-3: overnight, 6-maleimidohexanoic acid: 2 h; gradient: 20%–50% MeOH + 0.1% FA in 5 min, R_t_ = 3.7 min; MALDI-MS (*m/z*) for [M+H]^+^ (calculated): 4623.72 (4618.67); [M+2H]^2+^ (calculated): 2310.27 (2309.84). 

*H_2_N-CO-Lys-(PEG_3_)_2_-Mal_8_* **21**: Reaction times for conjugation of APG-1: 1 h, PEG_X_: 45 min, APG-2: 2 h, APG-3: overnight, 6-maleimidohexanoic acid: 2 h; gradient: 20%–50% MeOH + 0.1% FA in 5 min, R_t_ = 3.3 min; MALDI-MS (*m/z*) for [M+H]^+^ (calculated): 3631.08 (3630.11).

*H_2_N-CO-Lys-PEG_11_-(PEG_3_)_3_-Mal_8_* **22**: Reaction times for conjugation of APG-1: 1 h, PEG_X_: 45 min, APG-2: 2 h, PEG_Y_: 1 h, APG-3: overnight, PEG_Z_: 2 h, 6-maleimidohexanoic acid: 2 h; gradient: 30%–55% MeOH + 0.1% FA in 5 min, R_t_ = 3.3 min; MALDI-MS (*m/z*) for [M+Na]^+^ (calculated): 6970.43 (6970.00).

*H_2_N-CO-Lys-PEG_11_-(PEG_3_)_2_-Mal_8_* **23**: Reaction times for conjugation of APG-1: 1 h, PEG_X_: 45 min, APG-2: 2 h, PEG_Y_: 1 h, APG-3: overnight, 6-maleimidohexanoic acid: 2 h; gradient: 30%–55% MeOH + 0.1% FA in 5 min, R_t_ = 2.4 min; MALDI-MS (*m/z*) for [M+H]1+ (calculated): 4975.39 (4970.88).

*H_2_N-CO-Lys-PEG_11_-PEG_3_-PEG_1_-Mal_8_* **24**: Reaction times for conjugation of APG-1: 1 h, PEG_X_: 45 min, APG-2: 2 h, PEG_Y_: 1 h, APG-3: overnight, 6-maleimidohexanoic acid: 2 h; gradient: 30%–55% MeOH + 0.1% FA in 5 min, R_t_ = 2.3 min; MALDI-MS (*m/z*) for [M+H]^+^ (calculated): 4567.08 (4562.61).

*H_2_N-CO-Lys-PEG_5_-PEG_3_-PEG_1_-Mal_8_* **25**: Reaction times for conjugation of APG-1: 1 h, PEG_X_: 45 min, APG-2: 2 h, PEG_Y_: 1 h, APG-3: overnight, 6-maleimidohexanoic acid: 2 h; gradient: 20%–50% MeOH + 0.1% FA in 5 min, R_t_ = 3.4 min; MALDI-MS (*m/z*) for [M+H]^+^ (calculated): 4303.96 (4298.46).

*H_2_N-CO-Lys-PEG_11_-(PEG_1_)_3_-Mal_8_* **26**: Reaction times for conjugation of APG-1: 1 h, PEG_X_: 45 min, APG-2: 2 h, PEG_Y_: 1 h, APG-3: overnight, PEG_Z_: 2 h, 6-maleimidohexanoic acid: 2 h; gradient: 30%–55% MeOH + 0.1% FA in 5 min, R_t_ = 2.4 min; MALDI-MS (*m/z*) for [M+H]^+^ (calculated): 5526.38 (5519.07), [M+2H]^2+^ (calculated): 2760.11 (2760.04).

*H_2_N-CO-Lys-PEG_11_-(PEG_1_)_2_-Mal_8_* **27**: Reaction times for conjugation of APG-1: 1 h, PEG_X_: 45 min, APG-2: 2 h, PEG_Y_: 1 h, APG-3: overnight, 6-maleimidohexanoic acid: 2 h; gradient: 30%–45% MeOH + 0.1% FA in 5 min, R_t_ = 2.8 min; MALDI-MS (*m/z*) for [M+H]^+^ (calculated): 4361.71 (4358.48).

*H_2_N-CO-Lys-PEG_11_-(PEG_1_)_3_-Mal_16_* **28**: Reaction times for conjugation of APG-1: 1 h, PEG_X_: 45 min, APG-2: 2 h, PEG_Y_: 1 h, APG-3: overnight, PEG_Z_: 2 h, APG-4: overnight, 6-maleimidohexanoic acid: 2 h; gradient: 30%–55% MeOH + 0.1% FA in 5 min, R_t_ = 2.3 min; MALDI-MS (*m/z*) for [M+K]^+^ (calculated): 8469.42 (8470.84).

*H_2_N-CO-Lys-PEG_11_-PEG_3_-(PEG_1_)_2_-Mal_16_* **29**: Reaction times for conjugation of APG-1: 1 h, PEG_X_: 45 min, APG-2: 2 h, PEG_Y_: 1 h, APG-3: overnight, PEG_Z_: 2 h, APG-4: overnight, 6-maleimidohexanoic acid: 2 h; gradient: 30%–55% MeOH + 0.1% FA in 5 min, R_t_ = 2.4 min; MALDI-MS (*m/z*) for [M+K]^+^ (calculated): 8673.61 (8674.98), [M+2H]^2+^ (calculated): 4324.72 (4318.95).

*H_2_N-CO-Lys-PEG_11_-(PEG_3_)_2_-PEG_1_-Mal_16_* **30**: Reaction times for conjugation of APG-1: 1 h, PEG_X_: 45 min, APG-2: 2 h, PEG_Y_: 1 h, APG-3: overnight, PEG_Z_: 2 h, APG-4: overnight, 6-maleimidohexanoic acid: 2 h; gradient: 30%–55% MeOH + 0.1% FA in 5 min, R_t_ = 2.5 min; MALDI-MS (*m/z*) for [M+K]^+^ (calculated): 9086.1 (9083.25), [M+2H]^2+^ (calculated): 4527.44 (4523.09).

*H_2_N-CO-Lys-PEG_11_-(PEG_3_)_3_-Mal_16_* **31**: Reaction times for conjugation of APG-1: 1 h, PEG_X_: 45 min, APG-2: 2 h, PEG_Y_: 1 h, APG-3: overnight, PEG_Z_: 2 h, APG-4: overnight, 6-maleimidohexanoic acid: 2 h; gradient: 30%–55% MeOH + 0.1% FA in 5 min, R_t_ = 2.8 min; MALDI-MS (*m/z*) for [M+K]^+^ (calculated): 9914.00 (9899.80), [M+2H]^2+^ (calculated): 4938.16 (4931.36).

*H_2_N-CO-Lys-PEG_11_-PEG_5_-PEG_3_-PEG_1_-Mal_16_* **32**: Reaction times for conjugation of APG-1: 1 h, PEG_X_: 45 min, APG-2: 2 h, PEG_Y_: 1 h, APG-3: overnight, PEG_Z_: 2 h, APG-4: overnight, 6-maleimidohexanoic acid: 2 h; gradient: 30%–55% MeOH + 0.1% FA in 5 min, R_t_ = 2.6 min; MALDI-MS (*m/z*) for [M+K]^+^ (calculated): 9259.57 (9259.36), [M+2H]^2+^ (calculated): 4615.74 (4611.14).

*H_2_N-CO-Lys(DOTA)-PEG_11_-Mal_1_* **33**: gradient: 35%–60% MeOH + 0.1% TFA in 8 min, R_t_ = 5.1 min; yield: 2.9%; MALDI-MS (*m/z*) for [M+H]^+^ (calculated): 1324.60 (1324.73), [M+Na]^+^ (calculated): 1347.37 (1346.72), [M+K]^+^ (calculated): 1362.67 (1362.82).

*H_2_N-CO-Lys(DOTA)-PEG_11_-Mal_2_* **34**: Reaction times for conjugation of APG-1: 1 h, 6-maleimidohexanoic acid: 45 min; gradient: 35%–60% MeOH + 0.1% TFA in 8 min, R_t_ = 6.0 min; yield: 11.2%; MALDI-MS (*m/z*) for [M+H]^+^ (calculated): 1688.97 (1688.95), [M+Na]^+^ (calculated): 1711.39 (1710.93).

*H_2_N-CO-Lys(DOTA)-PEG_11_-PEG_1_-Mal_4_* **35**: Reaction times for conjugation of APG-1: 1 h, PEG_X_: 45 min, APG-2: 2 h, 6-maleimidohexanoic acid: 1 h; gradient: 45%–55% MeOH + 0.1% TFA in 8 min, R_t_ = 5.0 min; yield: 14.1%; MALDI-MS (*m/z*) for [M+H]^+^ (calculated): 2707.90 (2707.52).

*H_2_N-CO-Cys(S*-*tBu)-PEG_11_-PEG_1_-Mal_4_* **36**: Reaction times for conjugation of APG-1: 1 h, PEG_X_: 45 min, APG-2: 2 h, 6-maleimidohexanoic acid: 1 h; gradient: 45%–65% MeOH + 0.1% TFA in 8 min, R_t_ = 6.9 min; yield: 6.4%; MALDI-MS (*m/z*) for [M+H]^+^ (calculated): 2384.42 (2384.28), [M+Na]^+^ (calculated): 2406.62 (2406.27), [M+K]^+^ (calculated): 2422.40 (2422.37).

*H_2_N-CO-Lys-PEG_11_-PEG_1_-Mal_4_* **37**: Reaction times for conjugation of APG-1: 1 h, PEG_X_: 45 min, APG-2: 2 h, 6-maleimidohexanoic acid: 1 h; gradient: 30%–55% MeOH + 0.1% TFA in 8 min, R_t_ = 7.7 min; yield: 17.0%; MALDI-MS (*m/z*) for [M+H]^+^ (calculated): 2321.25 (2321.34).

*H_2_N-CO-Lys(DOTA)-PEG_11_-(PEG_1_)_2_-Mal_8_* **38**: Reaction times for conjugation of APG-1: 1 h, PEG_X_: 45 min, APG-2: 2 h, PEG_Y_: 1 h, APG-3: overnight, 6-maleimidohexanoic acid: 2 h; gradient: 30%–55% MeOH + 0.1% TFA in 8 min, R_t_ = 4.8 min; yield: 10.7%; MALDI-MS (*m/z)* for [M+H]^+^ (calculated): 4748.32 (4744.66), [M+2H]^2+^ (calculated): 2372.99 (2372.83).

*H_2_N-CO-Cys(S*-*tBu)-PEG_11_-(PEG_1_)_2_-Mal_8_* **39**: Reaction times for conjugation of APG-1: 1 h, PEG_X_: 45 min, APG-2: 2 h, PEG_Y_: 1 h, APG-3: overnight, PEG_Z_: 2 h, APG-4: overnight, 6-maleimidohexanoic acid: 2 h; gradient: 45%–65% MeOH + 0.1% TFA in 8 min, R_t_ = 6.7 min; yield: 13.0%; MALDI-MS (*m/z*) for [M+H]^+^ (calculated): 4424.95 (4421.42), [M+Na]^+^ (calculated): 4448.00 (4443.41), [M+K]^+^ (calculated): 4465.11 (4459.51). 

### 3.3. General Procedure for the Conjugation of Thiol-Bearing Synthons to the Multivalent Maleimide Scaffolds

Reaction in solution: To a solution of the respective maleimide-multimer (**35**, **36**, **38** or **39**, 0.46–2.5 μmol) in MeCN (100–400 μL) was added a solution of the respective thiol-bearing synthon to be multimerized (1-thio-β-D-galactose sodium salt, L-glutathione (reduced), c(RGDfC) or thiol-DOTA; at least 1.25 eq. per maleimide functionality) in phosphate buffered saline (0.1 M, pH 7.0, 300 µL), resulting in a pH of 6.8–7.2. After 5 min reaction time, the products were analyzed/isolated via HPLC and obtained as white solids or colorless oils after purification and lyophilization. Gradients used for HPLC analysis/purification and analytical data for each compound are given below. Reaction on solid phase: The resin containing the respective multivalent maleimide (**35** or **36**) was rinsed after the final maleimide conjugation with first MeCN and subsequently phosphate buffered saline (0.1 M, pH 7.0). The conjugation of thiol-bearing synthons was carried out by the addition of a solution of the respective thiol (at least 1.25 eq. per maleimide functionality) in phosphate buffered saline (0.1 M, pH 7.0, 500–750 µL) and incubation with the multivalent maleimide on solid support for 1h. The products were obtained by acidic cleavage from the resin and precipitation as described before and analyzed via HPLC. Gradients used for HPLC analysis/purification and analytical data for each compound are given below.

*Galactose_8_-DOTA* **40**: Gradient: 30%–55% MeOH + 0.1% TFA in 8 min, R_t_ = 4.3 min; yield: 22.8%; MALDI-MS (*m/z*) for [M+Na]^+^ (calculated): 6334.62 (6334.96), [M+2H]^2+^ (calculated): 3162.4 (3156.99).

*L-glutathione_8_-DOTA* **41**: Gradient: 30%–55% MeOH + 0.1% TFA in 8 min, R_t_ = 4.8 min; yield: 45.2%; MALDI-MS (*m/z*) for [M+Na]^+^ (calculated): 7222.99 (7223.31), [M+2H]^2+^ (calculated): 3601.52 (3600.16).

*c(RGDfC)_8_-DOTA* **42**: Gradient: 45%–65% MeOH + 0.1% TFA in 8 min, R_t_ = 4.3 min; yield: 19.8%; MALDI-MS (*m/z*) for [M+Na]^+^ (calculated): 9397.17 (9392.45), [M+2H]^2+^ (calculated): 4686.46 (4685.74).

*DOTA_8_-SS-tBu* **43**: Gradient: 20%–30% MeCN + 0.1% TFA in 8 min, R_t_ = 5.5 min; yield: 18.1%; MALDI-MS (*m/z*) for [M+Na]^+^ (calculated): 8156.95 (8149.09), [M+2H]^2+^ (calculated): 4068.7 (4064.06).

*L-glutathione_4_-DOTA* **44**: Gradient: 0%–100% MeCN + 0.1% TFA in 5 min, R_t_ = 1.8 min; MALDI-MS (*m/z*) for [M+H]^+^ (calculated): 3935.32 (3935.85).

*Galactose_4_-DOTA* **45**: Gradient: 0%–100% MeCN + 0.1% TFA in 5 min, R_t_ = 1.8 min; MALDI-MS (*m/z*) for [M+H]^+^ (calculated): 3491.79 (3491.68), [M+Na]^+^ (calculated): 3514.30 (3513.66), [M+K]^+^ (calculated): 3529.59 (3529.77), [M+Na+K]^+^ (calculated): 3552.07 (3551.75), [M+2K]^+^ (calculated): 3567.82 (3567.86).

*DOTA_4_-SS-tBu* **46**: Gradient: 0%–100% MeCN + 0.1% TFA in 5 min, R_t_ = 2.2 min; MALDI-MS (*m/z*) for [M+H]^+^ (calculated): 4239.60 (4237.12).

### 3.4. ^68^Ga-Radiolabeling of the DOTA-Comprising Multimers **40**–**43**

A solution of the respective DOTA-derivatized multimer (1.5–5 nmol) in tracepur H_2_O was added to 244–277 MBq of ^68^Ga^3+^ in a solution obtained by fractioned elution of a ^68^Ge/^68^Ga generator (Obninsk type, Eckert & Ziegler, Berlin, Germany) with HCl (0.1 M, 1.2 mL) and subsequent titration to pH 3.5–4.0 by addition of sodium acetate solution (1.25 M, 120–125 μL). After reaction for 10 min at 95 °C and cooling, the reaction mixtures were analyzed by analytical radio-HPLC. The radiolabeled products [^68^Ga]**40**, [^68^Ga]**42** and [^68^Ga]**43** were found to be ≥95% pure and obtained in specific activities of up to 147 GBq/µmol for **40**, up to 55 GBq/µmol for **42** and up to 163 GBq/µmol for **43**. 

## 4. Conclusions

It was shown that the synthesis of even complex dendritic scaffold structures based on acid-amide bonds on solid supports is possible using standard solid phase peptide synthesis protocols. By systematically investigating optimal building blocks and reaction parameters, the desired homogeneous and symmetric dendrons could be obtained. In addition, molecular designs deviating from the optimized structure could also be synthesized, enabling a highly modular dendron assembly on solid support. The obtained dendritic structures comprised up to 16 maleimide functionalities and could subsequently be efficiently reacted with structurally variable thiol-bearing bioactive molecules via click chemistry. Furthermore, some dendritic scaffolds were derivatized on solid support with the chelator DOTA and finally successfully radiolabeled with ^68^Ga after the multimerization reactions. This shows the applicability of the presented technique to the synthesis of multivalent radiotracers which can be used for molecular imaging purposes with PET.
